# Increase in (semi-) acute non-instrumented lumbar spine surgeries during the COVID-19 pandemic

**DOI:** 10.1016/j.bas.2025.104270

**Published:** 2025-05-02

**Authors:** C.L.C. Gallé, A.Y.J.M. Smeets, E.A. Krekels-Huijbregts, H. van Santbrink, R.H.L. Haeren

**Affiliations:** a Faculty of Health Medicine and Life Sciences, Maastricht University, Maastricht, the Netherlands; b Department of Neurosurgery, Maastricht University Medical Center+, Maastricht, the Netherlands; c Department of Neurosurgery, Zuyderland Medical Center, Heerlen, the Netherlands; d Department of Family Medicine, Maastricht University, Maastricht, the Netherlands; e CAPHRI School for Public Health and Primary Care, Maastricht University, Maastricht, the Netherlands; f School for Mental Health and Neurosciences, Maastricht University, Maastricht, the Netherlands

**Keywords:** COVID-19, Elective surgery, Spine surgery, Acute surgery, Surgical outcome

## Abstract

**Introduction:**

Due to the COVID-19 pandemic healthcare resources were reallocated, thereby reducing elective surgery capacity. An increase in acute surgeries due to postponed elective surgeries was expected. Since elective lumbar spine surgery for degenerative indications was among the cancelled or postponed surgical interventions, we hypothesized that the number of acute and semi-acute surgeries would increase during the pandemic.

**Research question:**

What was the effect of the COVID-19 pandemic on the number of (semi-)acute lumbar spinal surgeries?

**Material and methods:**

This prospective cohort study included patients who underwent non-instrumented degenerative lumbar spine surgery, i.e. interlaminar decompression, laminectomy or lumbar microdiscectomy. We distinguished a pre-COVID cohort (between 01.03.2019 and 29.02.2020) and a COVID-cohort (between 01.03.2020 and 28.02.2021).

**Results:**

We included 313 patients in the pre-COVID cohort and 194 in the COVID-cohort, reflecting a decline of 38.7 %. The number of (semi-)acute indications increased with 300 % in the COVID-cohort, which was mainly the result of more surgeries for severe and intractable radiculopathy. We also noted an increase in good clinical outcome and a decline in complications in the COVID-cohort.

**Discussion and conclusion:**

The number of elective degenerative lumbar spine surgeries declined due to the COVID-19 pandemic, while the rate of (semi)-acute lumbar spinal surgeries increased strongly. The latter may be due to altered patients’ clinical presentations and surgical decision making in times of severe health care scarcity of elective surgical care.

## Highlights

• The number of spinal surgeries declined with 38.7 % during the COVID-19 pandemic.
• (Semi-)acute spinal surgeries increased with 300 % in the COVID versus the pre-COVID cohort.
• More semi-acute lumbar spinal surgeries occurred due to severe, intractable radiculopathy.
• Increased rate of good surgical outcome and reduced complication rate during the COVID-pandemic.

## 1 Introduction

The COVID-19 pandemic has had a massive impact on elective surgeries around the world(Coulson et al., 2020). It was estimated that around 28.000.000 routine surgical procedures were postponed or cancelled globally during the 12 weeks of peak disruption in 2020 (Coulson et al., 2020). In the Netherlands, the number of non-oncological surgical procedures were reduced with around 30 %(de Graaff et al., 2022). This reduction in surgical procedures was mainly the result of reallocating personnel and equipment to the intensive care unit, to provide sufficient care for the large number of admitted COVID-19 patients. In addition, patients themselves cancelled their scheduled procedures because they were afraid of contracting COVID-19 in the hospital, or to limit the burden on the overloaded health system.

It was hypothesized that this deprioritization and cancellation of elective surgeries would lead to an increase in surgical waiting lists, clinical presentations in advanced stages of disease, and visits to the emergency department due to acute symptoms compared to the pre-COVID-19 period. As such, one would expect an increased number of (semi-)acute surgeries, dismal clinical outcomes, and inferior prognosis.

Elective lumbar spinal surgery for degenerative indications was among the numerous surgical interventions that were cancelled or postponed due to the COVID-19 pandemic. There are indeed many reports on the decreased rate of elective (lumbar) spinal surgeries due to the pandemic (Arnold et al., 2021; Blondel et al., 2023; Koruga et al., 2023; Norris et al., 2021; Oshima et al., 2023). Increased waiting lists for elective spinal surgery are also reported (Norris et al., 2021; Rathnayake et al., 2021). Potential negative effects of postponed elective lumbar spinal surgery are the development of increased pain, neurological deficits, in particular motor deficits, and cauda equine syndrome. This may result in an increased number of (semi-)acute surgical indications, with subsequent higher rates of unfavorable clinical outcomes. To date, there are no reports on the actual increase of (semi-)acute spinal surgeries during the COVID-19 pandemic, while studies on clinical outcomes and complication rates spinal surgeries did not observe major differences between pre-COVID- and COVID-periods (Jankovic et al., 2023; Riley and Verma, 2021).

Here, we hypothesized that the decline in elective lumbar spinal surgeries, due to the COVID-19 pandemic, would increase the rate of acute and semi-acute indications for surgery. Therefore, we performed a prospective cohort study of all lumbar spinal surgeries performed in two Dutch hospitals during the 12 months prior to the first COVID-19 lock down in the Netherlands, compared with the lumbar spinal surgeries in the 12 months following this first lock down.

## 2 Methods

### 2.1 Ethical considerations

This prospective cohort study was performed following approval by the Medical Ethics Committee Zuyderland & Zuyd (METCZ20210061) and the Medical Ethics Committee azM/UM (METC, 2021–2738). The requirement to obtain informed was waived in accordance with local legislation.

### 2.2 Patient selection

We included patients who underwent non-instrumented degenerative lumbar spine surgery, i.e. interlaminar decompression, laminectomy or lumbar microdiscectomy surgery, at the departments of Neurosurgery of Zuyderland Medical Center, Heerlen, and Maastricht University Medical Center (MUMC), Maastricht, the Netherlands. As the number of elective surgeries in both clinics were heavily restricted due to COVID-19 per March 2020, we included patients who were operated on between 1st of March 2019 and 1st of March 2021. This time window was selected to create two cohorts, both of one year duration: (1) the pre-COVID cohort (surgery between 01.03.2019 and 29.02.2020), and (2) the COVID-cohort (surgery between 01.03.2020 and 28.02.2021). According to the Dutch National Institute for Public Health and the Environment there have been two COVID-19 waves within the period of including our COVID-cohort (Ontwikkelingen).

Inclusion criteria encompass adult patients, i.e. 18 years and older, who underwent single- or multilevel non-instrumented degenerative lumbar spine surgery, with complete documentation of surgical indication, surgical procedure, complications and clinical outcome. Patients who underwent instrumented degenerative lumbar spine surgeries were excluded.

### 2.3 Outcome measures, data collection, and definitions

The primary outcome of this study is the difference in the number of acute and semi-acute surgeries between the pre-COVID and COVID-cohort. Secondary outcome parameters include the effects of COVID-19 pandemic on the preoperative duration of symptoms and on the outcomes and complications of surgery.

To this end, clinical data were extracted from the electronic patient files and included preoperative outpatient visits, clinical admission, and postoperative outpatient visits up to one year after surgery. The following baseline data were collected: age, sex, body mass index (BMI), American Society of Anesthesiologists (ASA) classification, indication for surgery, previous lumbar spine surgery, syndrome of preoperative symptoms, preoperative duration of symptoms (in weeks), number of preoperative neurosurgical consultations, duration on the surgical waiting list (in weeks), duration between last outpatient visit and surgery (in weeks). For syndrome of preoperative symptoms, we distinguished the following indications: lumbosacral radicular syndrome, neurogenic claudication, neurological motor deficits and cauda equina syndrome. All data was collected by one author (C.G.), and when in doubt, one or more senior neurosurgeons (A.S., H.v.S., or R.H.) were consulted to define the final interpretation of the data.

Regarding surgical treatment, we registered the type of intervention (i.e. interlaminar decompression, laminectomy, or microdiscectomy), timing of the surgery (i.e. acute, semi-acute or elective), extension of surgery (i.e. single level or multilevel), and level of surgery, (i.e. L1-L2, L2-L3, L3-L4, L4-L5, and/or L5-S1). We differentiated interlaminar decompression from laminectomy based on partial or complete removal of the lamina, respectively. The timing of surgery was defined as acute when surgery was performed within 24 h after clinical presentation. Indications for Acute and semi acute surgery were clearly defined. Acute surgery was performed for a cauda equina syndrome that was present since less than 24 h or for major or progressive motor deficits that developed within the last 3 days before presentation. Semi-acute surgery was defined as surgery performed between 24 h and one week after clinical presentation. Indications for semi-acute surgery included: cauda equina syndrome present longer than 24 h, neurological motor deficits that were present longer than 3 days and not progressive, or severe and medically intractable pain. Elective surgery was recognized in case the patient was scheduled from the regular waiting list in the absence of acute or semi-acute indications.

Lastly, we collected follow-up data including duration of follow-up, effect of surgery on the preoperative symptoms (i.e. good or poor), and complications (i.e. new neurological deficits, recurrent surgery, wound infection, treatment failure, postoperative bleeding, and cerebrospinal fluid (CSF) fistula/leakage. Good clinical outcome on preoperative symptoms was defined as improvement or disappearance of symptoms, while stabilization or deterioration of symptoms was considered as poor clinical outcome. Due to the exceptional organizational conditions, common patient-reported outcome measures such as the Visual Analogue Scale or the Oswestry Disability Index were not available for our patients. In the absence of these solid outcome measures, we opted for the binary ‘good or poor’ clinical outcome measure. Interpretation and selection of ‘good’ or ‘poor’ was based on the notes in the electronic patient files at postoperative outpatient clinic visits. This strategy may have resulted in an interpretation bias. Interpretation and selection of ‘good’ or ‘poor’ was based on the notes in the electronic patient files at postoperative outpatient clinic visits. This interpretation and selection was performed by one author (C.G.). When in doubt senior neurosurgeons (A.S., H.v.S. or R.H.) were consulted to define whether the clinical outcome was good or poor.

### 2.4 Study sites

Patients were included from two study sites, i.e. Zuyderland Medical Center, Heerlen, the Netherlands and MUMC+, Maastricht, the Netherlands. The Zuyderland Medical Center is a regional hospital, with a particular focus on spinal neurosurgery, whereas the MUMC+ is an academic hospital with less focus on spinal neurosurgery. Neurosurgeons from the department of neurosurgery of the MUMC + are working on secondment base at the Zuyderland Medical Center. Hence, the same neurosurgeons practice in both centers, limiting the risk of surgeons bias between the two centers. The catchment area of the two study sites includes the whole Southern area of the region of Limburg encompassing around 600.000 inhabitants. In general, a summed total of around 300–350 non-instrumented degenerative lumbar spine procedures are performed in the two centers each year.

### 2.5 Statistical analyses

Statistical analyses were carried out using IBM SPSS statistics 29. Data were extracted by three researchers (CG, AS, RH). Patient characteristics, preoperative characteristics, surgical characteristics and clinical outcomes were compared between the pre-COVID and COVID-cohort. Descriptive data were generated for all variables. Continuous variables are described as means with standard deviations and proportions are noted as percentages. The independent *t*-test was used to analyze means. Chi-square test and Fisher's exact test were used for the categorial variables with two categories. For multiple categories, independent-samples proportions procedure was used for comparison of proportions between groups. A multiple logistic regression analysis was performed, including the surgical characteristics that were seen as clinically relevant as potential confounders. We analyzed the difference of semi-acute and acute surgeries versus elective surgeries in the pre-COVID versus COVID cohort. A p-value below 0.05 was considered statistically significant. Missing data were excluded from the analyses per variable.

## 3 Results

### 3.1 Patient characteristics

**Fig. 1:**
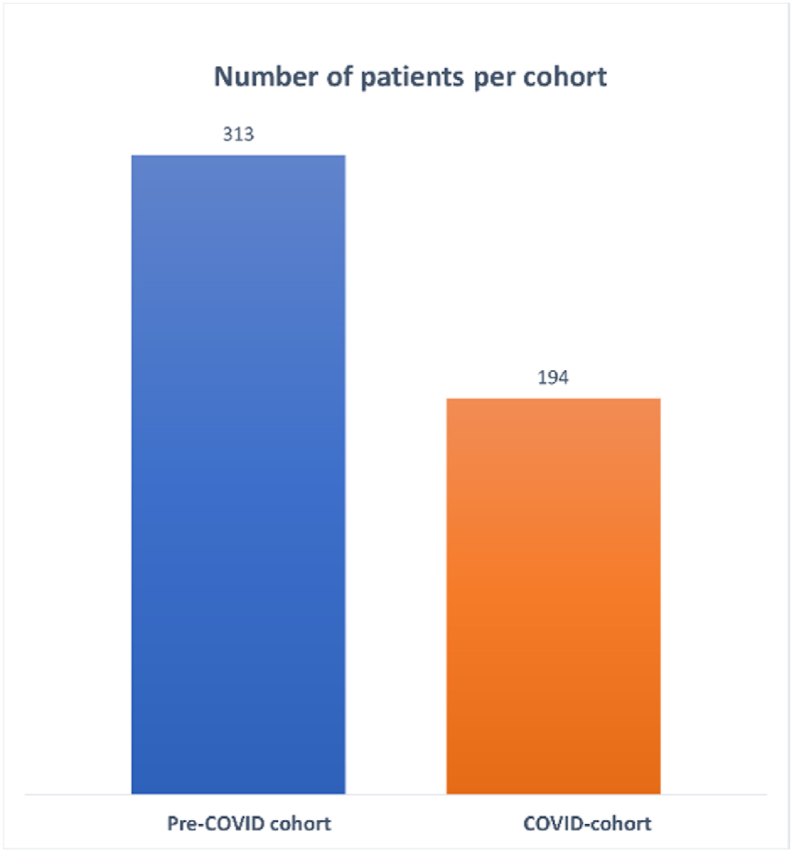
Depiction of the total number of patients per cohort. On the left are the number of patients of the pre-COVID cohort, in blue. On the right are the number of patients of the COVID cohort, in orange.

We identified 507 patients who met our inclusion and exclusion criteria. Of these, 341 patients were operated at Zuyderland Medical Center, and 166 patients at MUMC+. In the pre-COVID year, 313 patients underwent non-instrumented lumbar spine surgery compared to 194 patients in the first COVID-year, which is a reduction of 38.0 % (Fig. 1). Patient characteristics such as age, sex, BMI and ASA classification were comparable for the pre-COVID and COVID-cohort, with a mean age of around 65 years, between 46 and 47 % female patients, a mean BMI of around 28 kg/m^2^, and a mean ASA classification of 2. More detailed patient characteristics can be found in Table 1.

**Table 1:** Patient characteristics **Abbreviations:** N = number; SD = standard deviation.

Variable	All patients (n = 507)	Pre-covid cohort (n = 313)	COVID-cohort (n = 194)	p-value
**Hospital**				
Zuyderland (N, %)	341 (67.3 %)	195 (62.3 %)	146 (75.3 %)	***<0.01***
MUMC+ (N, %)	166 (32.7 %)	118 (37.7 %)	48 (24.7 %)	***<0.01***
**Age** (mean, SD) years	65.2 (13.2)	65.2 (13.2)	65.0 (13.2)	0.841
**Sex** (N, %) Female	236 (46.5 %)	144 (46.0 %)	91 (46.9 %)	0.867
**BMI** (mean, SD) kg/m^2^	28.3 (4.5)	28.1 (4.4)	28.6 (4.7)	0.158
**ASA-classification score**				
1 (N, %)	66 (13.0 %)	46 (14.7 %)	20 (10.3 %)	0.150
2 (N, %)	293 (57.9 %)	183 (58.7 %)	110 (56.7 %	0.665
3 (N, %)	145 (28.7 %)	81 (26.0 %)	64 (33.0 %)	0.089
4 (N, %)	2 (0.4 %)	2 (0.6 %)	0 (0 %)	0.264
**Duration of symptoms (in weeks)**				
Elective patients (mean, SD)	76.1 (107.5)	68.4 (71.1)	90.0 (152.3)	***0.04***
Acute patients (mean, SD)	28.2 (44.1)	12.9 (22.8)	38.4 (51.7)	***0.03***
**Duration on waiting list (in weeks)**				
Elective patients (mean, SD)	8.3 (6.1)	7.6 (5.1)	9.7 (7.4)	***<0.01***
Acute patients (mean, SD)	6.1 (21.4)	8.1 (32.5)	4.9 (9.2)	0.65
**Number of preoperative outpatient visits** (mean, SD)	1.5 (1.1)	1.5 (1.1)	1.5 (0.9)	0.85
**Previously on waiting list***(only for acute patients)*				
Number of patients (N, %)	3 (5.5 %)	0 (0 %)	3 (8.6 %)	0.10

The mean duration of symptoms in the pre-COVID cohort was 68.4 ± 71.1 weeks which was shorter (p = 0.04) than the 90.0 ± 152.3 weeks in the COVID-cohort. The duration on the waiting list increased significantly for the COVID-cohort compared to pre-COVID cohort (9.7 ± 7.4 vs. 7.6 ± 5.1 weeks respectively, p < 0.01). Notably, the duration on the waiting list increased only for the electively scheduled patients in the COVID-cohort (Table 1). Among the acutely operated patients in the COVID-cohort, three were already on our waiting list, compared to no patients in the pre-COVID cohort.

### 3.2 Timing of surgery and surgical characteristics

**Fig. 2:**
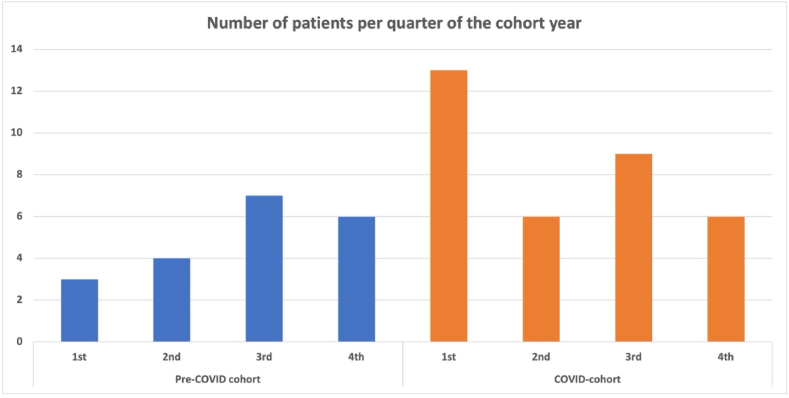
This shows the number of acute and semi-acute cases per cohort, with the pre-COVID cohort on top and the COVID cohort on the bottom. The bars are separated in the number of acute (gray) and semi-acute (pink) cases.

Surgery in the acute or semi-acute setting was indicated in 20 out of the 313 patients (6.4 %) in the pre-COVID cohort compared to 35 out of the 194 (18.0 %) patients in the COVID-cohort. In absolute numbers, this reflects an increase of 75 % (p < 0.0001), while this a relative increase of almost 300 % (Fig. 2). Among these patients, nine (2.9 %) patients in the pre-COVID cohort compared to 13 (6.7 %) in the COVID-cohort underwent surgery for an acute indication (Table 2). In almost all these cases, the acute indication was a cauda equine syndrome. In one case of the COVID-cohort, the indication for acute surgery was progressive neurological motor deficits. In addition, a total of 11 (3.5 %) patients were indicated for semi-acute surgery in the pre-COVID cohort, compared to 22 (11.3 %) in the COVID-cohort. Of these semi-acute cases, the indication was neurological motor deficits in 9 of 11 patients in the pre-COVID cohort, compared to 5 of 22 patients in the COVID-cohort. The remaining semi-acute indications were severe and medically intractable pain, and one case within the pre-COVID cohort presented with a cauda equina syndrome that was already present for more than a week.

**Table 2:** Surgical characteristics **Abbreviations:** ILD = interlumbar decompression; N = number.

Variable	All patients (n = 507)	Pre-covid cohort (n = 313)	COVID-cohort (n = 194)	p-value
**Setting**				
Elective (N, %)	452 (89.2 %)	293 (93.6 %)	159 (82.0 %)	***<0.01***
Acute/Semi-acute (N, %)	55 (10.8 %)	20 (6.4 %)	35 (18.0 %)	***<0.01***
*Acute* (N, %)	22 (4.3 %)	9 (2.9 %)	13 (6.7 %)	***0.04***
*Semi-acute* (N, %)	33 (6.5 %	11 (3.5 %)	22 (11.3 %)	***<0.01***
**Indication**				
Neurogenic claudication (N, %)	325 (64.4 %)	198 (63.5 %)	127 (65.8 %)	0.74
Radiculopathy (N, %)	144 (28.4 %)	96 (30.8 %)	48 (24.9 %)	0.15
Neurological motor deficit (N, %)	14 (2.8 %)	8 (2.6 %)	6 (3.1 %)	0.61
Cauda equine syndrome (N, %)	22 (4.4 %)	10 (3.2 %)	12 (6.2 %)	0.14
**Intervention**				
ILD (N, %)	286 (56.4 %)	169 (54.0 %)	117 (60.3 %)	0.14
Laminectomy (N, %)	70 (13.8 %)	47 (15.0 %)	23 (11.9 %)	0.29
Discectomy (N, %)	151 (29.8 %	97 (31.0 %)	54 (27.8 %)	0.45
**Number of operated levels**				
One level (N, %)	420 (82.8 %)	260 (83.1 %)	160 (82.5 %)	0.88
Two levels (N, %)	70 (13.8 %)	40 (12.8 %)	30 (15.5 %)	0.42
Three or more levels (N, %)	17 (3.4 %)	13 (4.2 %)	4 (2.1 %)	0.16
**Operated lumbar level***(only for single level surgery)*				
L1-L2 (N, %)	6 (1.2 %)	6 (2.3 %)	0 (0.0 %)	0.06
L2-L3 (N, %)	46 (9.1 %)	26 (9.9 %)	20 (12.4 %)	0.47
L3-L4 (N, %)	79 (15.7 %)	47 (17.9 %)	32 (19.9 %)	0.70
L4-L5 (N, %)	233 (46.2 %)	145 (55.3 %)	88 (54.7 %)	0.78
L5-S1 (N, %)	59 (11.7 %)	38 (14.5 %)	21 (13.0 %)	0.62
**Previous surgery on same level**				
Number of patients (N, %)	56 (11.1 %)	33 (10.6 %)	23 (11.9 %)	0.68

An analysis was conducted to evaluate differences in waiting list duration and symptom duration per indication across the cohorts. This analysis revealed no statistically significant differences, except for the symptom duration for the indication radiculopathy. In this subgroup, a significant difference was observed between the pre-COVID and COVID cohorts, with mean symptom durations of 49 ± 47 weeks and 62 ± 55 weeks, respectively (p = 0.012, Table 3).

**Table 3:** Waiting time per surgical indication.

Variable		All patients (n = 507)	Pre-covid cohort (n = 313)	COVID-cohort (n = 194)	p-value
**Duration on waiting list in weeks (mean, SD)**	*Radiculopathy*	6.20 (4.76)	5.70 (3.76)	7.06 (6.28)	0.106
	*Neurological deficits*	1.85 (4.86)	12.86 (20.89)	68.67 (152.61)	0.586
	*Cauda syndrome*	0.59 (1.01)	0.30 (0.67)	0.83 (1.19)	0.205
	*Neurogenic claudication*	9.76 (10.49)	9.26 (11.62)	10.46 (8.22)	0.278
**Symptom duration in weeks (mean, SD)**	*Radiculopathy*	48.57 (47.39)	41.68 (41.94)	62.64 (54.76)	0.012∗
	*Neurological deficits*	38.62 (103.72)	1.25 (2.18)	2.83 (6.46)	0.356
	*Cauda syndrome*	12.05 (20.05)	5.1 (5.8)	17.83 (25.67)	0.142
	*Neurogenic claudication*	86.29 (121.17)	80.75 (78.50)	94.04 (166.31)	0.337

**Abbreviations:** N = number; SD = standard deviation.

We also evaluated the number of (semi-)acute cases over time during both cohorts. We observed an increase in the number of (semi-)acute cases (n = 14) in the first three months of the COVID-19 pandemic compared to the number of (semi-)acute cases (ranging from 4 to 8 cases per quarter) during the other quarters of both cohorts.

In general, the indications for elective surgeries, the rate of single – or multilevel surgery, the operated levels, as well as the numbers of recurrence surgery were comparable for the pre-COVID and COVID-cohort (Table 2).

The surgical characteristics presented in Table 2 were included in a multiple logistic regression analysis, shown in Table 4. This analysis revealed that the COVID-19 pandemic, along with the indications radiculopathy, neurological deficits, and cauda equina syndrome, were significantly associated with an increased odds of (semi-)acute surgical intervention.

**Table 4:** Multiple logistic regression.

Variable		p-value	OR	95 %CI
**Cohort**	Covid	<0.001	5.13	2.25–11.68
**Indication**	Radiculopathy	<0.001		
	Neurological deficits	<0.001	32.55	7.31–145.03
	Cauda Equina	<0.001	77.14	18.01–330.50
	Neurogenic Claudication	0.347	0.61	0.22–1.70
**Intervention**	ILD	0.428		
	Discectomy	0.216	1.86	0.70–4.96
	Laminectomy	0.449	1.59	0.48–5.27
**Level of decompression**	L1	0.900		
	L2	0.895	0.76	0.01–44.85
	L3	0.644	0.38	0.01–22.30
	L4	0.881	0.74	0.02–37.63
	L5	0.704	0.46	0.01–26.06
	S1	0.999	a	0.00
**Previous surgery on same level**		0.309	1.70	0.61–4.76
**Multiple level**		0.999	a	0.00

**Abbreviations:** N = number; SD = standard deviation; CI = confidence interval.

∗∗This data is not available for reference variables.

a Due to low number of cases unreliable result.

### 3.3 Clinical outcomes and complications

A good clinical outcome following surgery was significantly (p < 0.01) more frequent in the COVID-cohort (87.1 %) than in pre-COVID cohort (77.6 %). The postoperative development of new symptoms, in particular radiculopathy, was slightly more common in the pre-COVID cohort (8.8 %) than in the COVID-cohort (6.0 %). The complication rate was also higher in the pre-COVID cohort than the COVID-cohort (18.3 % versus 11.6 %, respectively, p = 0.04), which was mainly related to the higher rate of treatment failure (12.7 % versus 5.3 %, respectively). The number of wound infections or other complications such as CSF fistula/leakage was similar for both cohorts. The mean number of postoperative outpatient visits and the duration of postoperative follow-up did also not differ (Table 5).

**Table 5:** Clinical outcome.

Variable	All patients (n = 507)	Pre-covid cohort (n = 313)	COVID-cohort (n = 194)	p-value
**Good clinical outcome** (N, %)	398 (82.9 %)	229 (77.6 %)	169 (91.4 %)	***<0.01***
**New postoperative symptoms**				
None (N, %)	445 (92.3 %)	271 (91.2 %)	174 (94.1 %)	0.26
Neurological deficit (N, %)	24 (5.0 %)	19 (6.4 %)	5 (2.7 %)	0.07
(Pseudo)radiculopathy (N, %)	2 (0.4 %)	1 (0.3 %)	1 (0.5 %)	0.74
Other	11 (2.3 %)	6 (2.0 %)	5 (2.7 %)	0.63
**Postoperative complications**				
Number of complications	77 (15.7 %)	55 (18.3 %)	22 (11.6 %)	***0.04***
Wound infection (N, %)	12 (15.6 %)	7 (12.7 %)	5 (22.7 %)	0.84
Failure of treatment (N, %)	48 (62.3 %)	38 (69 %)	10 (45.5 %)	***<0.01***
Other (N, %)	17 (22.0 %)	10 (18.2 %)	7 (31.8 %)	0.84
**Duration of follow-up**				
Number of weeks (mean, SD)	17.3 (19.3)	18.1 (22.6)	15.9 (15.6)	0.12

**Abbreviations:** N = number; SD = standard deviation.

## 4 Discussion

In this study, we found that the number of non-instrumented lumbar spinal surgeries for degenerative indications declined with 38.7 % during the first year of the COVID-19 pandemic, while the duration of symptoms and the duration on the waiting list increased. Simultaneously, the number of (semi)-acute surgeries increased with 300 % compared to the pre-COVID cohort, which was mainly the result of more semi-acute surgeries based on the indication of severe and medically intractable radiculopathy. We observed an increased rate of good clinical outcome and a decline in the complication rate in the COVID-cohort, while the infection rate remained stable.

### 4.1 General effects of COVID-19 pandemic on elective lumbar spinal surgeries

In our hospitals, the number of elective surgeries declined heavily (38.7 %) in the first year of the COVID-19 pandemic. This was mainly the result of reduction in the operation room capacity. During the first COVID-19 wave, these reductions have peaked up to 70–80 % in our centers. As a result, operation rooms were solely used for surgical procedures with high priority, such as direct life-threatening and oncological surgery, thereby heavily affecting the number of surgeries of lower priority such as elective lumbar spinal surgery. Specified for our group of neurosurgeons we prioritized life threatening life threatening and oncological surgery, such brain trauma and glioma surgery, were prioritized, followed by rapidly progressive neurological disorders, such as myelopathies, and specific surgeries of the key topics of our neurosurgical department, such as epilepsy surgery. Consequently, the mean duration on the waiting lists increased from 7.6 to 9.5 weeks for our elective lumbar spine patients. The duration on the waiting lists would have been even higher if there was not a simultaneous decrease in the number of outpatient visits. This decrease was the result of nationwide contact restrictions heavily limiting outpatient visits, and of patients’ anxiety to contract a COVID-19 infection during outpatient/hospital visit. A decline in neurosurgical referrals during COVID-19 has been described previously, we could not confirm this due to the retrospective design of the study and therefore the unavailability of the data. (Jayakumar et al., 2020; Koester et al., 2021). But in our series, this effect may be reflected by an increase in the mean duration of symptoms from 64.7 to 80.7 weeks as reported at the first outpatient visit.

Regarding the decline in surgical spinal procedures, our result is comparable to the 34.4 % decline reported by Tarawneh et al. who analyzed the decline in elective non-instrumented lumbar laminectomy procedures for degenerative indications in multiple databases in the United States of America from March 2020 to May 2021 (Tarawneh et al., 2023). Our decline in elective lumbar spine surgeries is however higher than the 6.9 % and 22.1 % decline reported by Japanese spine and Croatian neurosurgical centers, respectively, over the year 2020 (Koruga et al., 2023; Oshima et al., 2023).This may be due to fact that these studies described all types of spine surgery in their series, including indications that are prioritized over elective lumbar spinal surgery, such as traumatic spine surgery as well as cervical and thoracic spine surgery for myelopathy. Moreover, these series included the year 2020 as a whole to compare with, whereas we compared a one-year cohort from the start of the first COVID-19 wave, i.e. March 2020. In the first two months of 2020, there were barely any restrictions and surgical reductions due to COVID-19 which may have limited the decline in spinal surgical procedures in these series as well.

### 4.2 Increase in (semi-)acute surgeries during COVID-19 pandemic

We found an evident increase of 300 % in the number of (semi-)acute surgeries in our COVID-cohort compared to the pre-COVID cohort. This increase is in line with our expectation that cancelled and postponed lumbar spinal surgeries would lead to an increase in (semi-)acute surgeries. We found one other study describing an increase in the number of acute indications of spine surgery from 19.0 % to 29.1 %, for a pre-COVID and COVID-cohort respectively(Jankovic et al., 2023). Here, all locations and types of spine surgery, including cervical and thoracic levels as well as traumatic and infection etiology, were included and an acute indication was defined as “requiring timely treatment within 24 h”. This makes this patient series only limitedly comparable to our series, yet the increase in acute cases is noteworthy.

The increase in (semi-)acute cases in our series was mostly the result of a higher rate of severe and medically intractable pain indications. The number of surgeries for cauda equina syndrome as well, as neurological motor deficits, also increased significantly, yet in absolute numbers the increase was only 4 patients. On the one hand, these findings suggests that prolonged duration of symptoms until surgery may lead to neurological deterioration including cauda equina symptoms as well as motor deficits. On the other hand, the findings may suggest that prolonged duration of symptoms may lead to an increase in the number of patients that experience severe and medically intractable radiculopathy. We deem this increase can be explained by factors that are patient-related as well as physician-related in times of the COVID-19 pandemic. The multiple logistic regression analysis confirmed that the COVID-19 period was a significant factor associated with the rise in (semi-)acute surgical cases. Among the other variables analyzed, only the surgical indications of radiculopathy, neurological deficits, and cauda equina syndrome were identified as statistically significant contributors. This finding may support the hypothesis that indications associated with severe pain, particularly semi-acute conditions, were more frequently observed during the pandemic. This interpretation is further supported by the finding that, among all indications, only the symptom duration for patients with radiculopathy was significantly longer in the COVID cohort. This suggests that the delay was more likely due to patients presenting later to their healthcare provider, rather than an extended waiting list duration.

Regarding the patient-related factors, the COVID-19 pandemic itself and worldwide contact restriction measures had strong effects on the mental health of patients. According to the World Health Organization, the rate of depression and anxiety among the general population increased rapidly in the first year of the pandemic, and its prevalence had risen up to 35 % during the pandemic (Delpino et al., 2022; World Health Organization (WHO), 2022; Invloed van de corona). Depression and anxiety may lead to catastrophizing of clinical conditions and increased complaining about worsening of symptoms(Held et al., 2022). Moreover, the prolonged duration on the waiting list, and thus prolonged suffering, as well as the uncertainty about a date for surgery, may have particularly affected the mental health of these patients. Lastly, physical and social activities were reduced heavily due to nationwide restrictions during the COVID-19 pandemic. Physical activity may decrease pain perception (Law and Sluka, 2017), while social interactions may also decrease perceived pain(Krahé et al., 2013). As a result, these patient-related factor may (partly) explain the increased number of patients that were operated for severe and intractable radiculopathy in a semi-acute setting during the COVID-19 pandemic.

Physicians were confronted with altered logistics of the healthcare system. As a result of strong reductions in operation rooms, prioritization of patients for surgery was required. As mentioned, elective lumbar spinal surgery was prioritized relatively low compared to other neurosurgical pathologies leading to an enormous scarcity of surgical capacity and uncertainty for elective lumbar spinal surgery. While confronted with this scarcity and uncertainty, it is plausible that physicians changed their criteria and thresholds for semi-acute surgery, consciously or unconsciously. By lowering the thresholds for semi-acute surgery for severe and intractable pain, physicians were able to get patients operated despite the limited surgical capacity. The increase in (semi-)acute cases may also be attributed to a shift in classification rather than a change in patient needs.

**Fig. 3:**
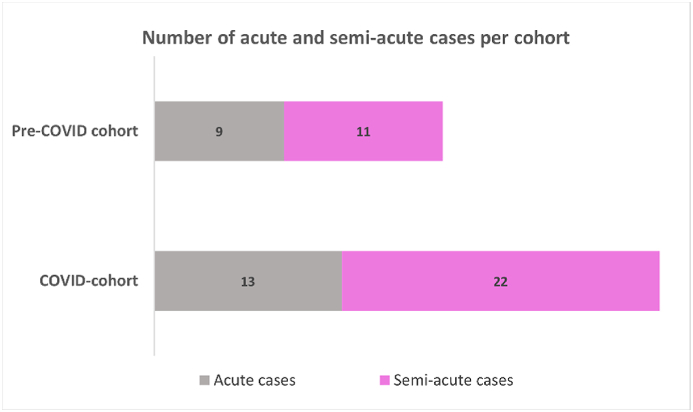
This figure visualizes the number of (semi-)acute cases per quarter of the pre-COVID (blue) and COVID (orange) cohort.

Reduction in operation room capacity was highest during the first COVID-19 wave which is reflected by the first three months of our COVID-cohort. During this time period, we noted a sharp increase in indicated (semi-)acute cases (Fig. 3). We consider it likely that this increase exemplifies the aforementioned patients' and physicians' related factors in response to the COVID-19 pandemic. Moreover, the duration and progression of the COVID-19 pandemic was still uncertain at that point. This uncertainty may have further aggravated patients behavior and expectations as well as physician's altered indication behavior.

### 4.3 Clinical outcome and complications

In our series, the rate of good clinical outcome increased from 77.6 % in the pre-COVID cohort to 87.1 % in the COVID-cohort. It is important to underline that the assessment of clinical outcome in our series was based on the notes at postoperative outpatient visits, and not based on more solid patient-reported outcome measures. This may have led to an interpretation bias. Nevertheless, we compared our findings to previous studies, and found only one other study evaluating clinical outcome of spinal procedures in a pre- and post-pandemic cohort. In this study, including all types of spine surgery, no differences were found in clinical outcome (Oshima et al., 2023). This contrasts our findings, which were unexpected, in particular given the multiple factors that negatively affect clinical outcome of spinal surgery. As mentioned, there was a rapid and steep increase in mental health problems due to the COVID-19 pandemic. Mental health problems, especially depression, are reported to be associated with more severe symptoms and disability postoperatively (Held et al., 2022). Additionally, we noted an increase in the duration of preoperative symptoms as well as of the surgical waiting lists. Patients with prolonged symptoms prior to their surgery have been shown to experience smaller improvements in postoperative leg pain and quality of life (Kasir et al., 2023).

Despite all this, we noted an increase in good clinical outcome. We speculate that two factors may have contributed to this finding. Firstly, we assume that our neurosurgeons have set indications for surgery more strictly as a result of the limited operation room capacity The increase from 6 % to 18 % of more robust (semi)-acute indications supports this assumption. Alternatively, adjusting indications may have resulted in a selection bias favoring patients with the highest likelihood of a favorable surgical outcome. Secondly, higher severity of preoperative symptoms as well as lower preoperative quality of life have been described to increase the clinical outcomes of lumbar discectomy surgeries (Rushton et al., 2018). We were not able to assess pre- and postoperative pain, disability and quality scores in this study. Yet, the higher number of patients operated on for severe and intractable pain as well as probable increased rate of mental health problems among patients and COVID-19 pandemic measures make it plausible that patients in our COVID-cohort experienced more severe symptoms and reduced quality of life.

Notably, we also found a decline in perioperative complications. This decline was mainly the result of treatment failure, which we included as a complication. The lower rate of treatment failure in the COVID-cohort is likely explained similarly as the increased rate of good clinical outcome as described previously. Although one may expect increased infection rates associated with the hospital's high number of COVID-19 patients, the rate of infection did not increase in our series. Jankovic et al. also reported a decline in perioperative complications in a pandemic cohort compared to a pre-pandemic cohort of patients who underwent spine surgery(Jankovic et al., 2023). The authors hypothesized this was mainly the result of changes in patients' characteristics and co-morbidities. Although we did not include co-morbidities in our series, general and health characteristics such as sex, age, BMI and ASA-classification were comparable between the cohorts.

### 4.4 Strengths and limitations

Our study has several strengths. This is the first study to evaluate the effects of the COVID-19 pandemic on the volume, outcome and complications of elective as well as (semi)-acute surgery for degenerative lumbar spine indications. As a result of our catchment area, our cohort is relatively large making statistical analyses more powerful.

Our study also has limitations. This study is based on data extraction from electronic patient file system which may introduce an interpretive selection bias and the frequent use of dichotomous outcomes. Specifically, clinical outcome (good or poor) was solely based on the interpretation of postoperative outpatient clinic’ notes, making this outcome measure less robust and related conclusions more speculative. Lastly, surgeries were indicated and performed by one group of neurosurgeons in the Southern region of the Netherlands. While this approach minimizes variability in surgical decision-making, it also limits external validity and generalizability of the findings.

## 5 Conclusions

During the first year of the COVID-19 pandemic, an increase in the rate of (semi)-acute lumbar spinal surgeries was noted in comparison to the year pre-COVID, while a lower number of elective non-instrumented degenerative lumbar spinal surgeries were performed. The reasons for this increase are speculative, yet may be due to changes in patients’ clinical presentations, and physician attitudes on surgical decision making in times of strict health care regulation and limited capacity. To study the effect of scarcity in operating room capacity, e.g. due to a pandemic, on patient outcome, in spine surgery more studies are needed.

## Ethical approval

Ethical approval was obtained through the responsible ethical committee (Medisch-ethische toetsingscommissies (METC)) for the protocol of this study (Ref. nr 2021–2738).

## Author's contributions

EK, RH, AS developed the protocol for this study and performed patient inclusion. RH, AS, CG and EK performed to the acquisition of data. RH, AS, CG and HS contributed to analysis and interpretation of data. RH and CG wrote the main manuscript and prepared the tables and figures. All authors reviewed and provided final approval of the manuscript.

## Ethical approval

Ethical approval was obtained through the responsible ethical committee (Medisch-ethische toetsingscommissies (METC)) for the protocol of this study (Ref. nr 2021–2738).

## Availability of data and materials

Not applicable.

## Funding

Not applicable.

## Competing interests

Authors have no competing interests as defined by Springer, or other interests that might be perceived to influence the results and/or discussion reported in this paper.
